# Impact of surgical timing on chronic subdural hematoma outcomes: novel insights from a multicenter study

**DOI:** 10.1007/s10143-025-03502-4

**Published:** 2025-04-03

**Authors:** Stefano Colonna, Enrico Lo Bue, Alessandro Pesaresi, Lorenzo Dolci, Andrea Gatto, Luca Ceroni, Alessandro Pesce, Maurizio Salvati, Daniele Armocida, Alessandro Frati, Antonio Santoro, Alice Mistretta, Diego Garbossa, Fabio Cofano

**Affiliations:** 1 Department of Neuroscience “Rita Levi Montalcini”, Neurosurgery Unit, University of Turin, Via Cherasco, 15, Turin, 10126 Italy; 2 Department of Psychology, University of Turin, Turin, Italy; 3 Department of Neurosurgery, University of Rome “Tor Vergata”, Rome, Italy; 4 Department of Neurosurgery, A.O. Ospedale Maggiore Parma, Parma, Italy; 5 Neurosurgery, IRCCS Neuromed, Pozzilli, Italy; 6 Human Neurosciences Department, Neurosurgery Division, “Sapienza” University, Rome, Italy; 7 Department of Intensive care unit and Emergency, CTO Hospital, A.O.U. “Città della Salute e della Scienza, Turin, Italy

**Keywords:** Chronic subdural hematoma, Surgical timing, Mild symptomatic, Multicenter study

## Abstract

**Objective:**

Chronic Subdural Hematoma (CSDH) is one of the most frequently encountered conditions in the neurosurgical practice. The role of timing in CSDH surgery in mild symptomatic patients remains uncertain. The aim of this study was to analyze the prognostic role of surgical timing in patients with mild symptomatic CSDH.

**Methods:**

In this multicenter retrospective study, patients diagnosed with mild symptomatic CSDH who underwent surgical evacuation were enrolled. Marwalder Grading System (MGS) and GCS scores were used for neurological evaluation. Patients presenting with preoperative GCS score ≥ 13 and MGS score ≤ 2 scores were defined as “mild symptomatic”. A ROC curve analysis was used to identify the optimal surgical timing associated with favorable postoperative outcome. Univariate and multivariate analysis were used to verify the association between surgical timing and postoperative neurological outcome, length of hospitalization, and postoperative complication.

**Results:**

A total of 160 patients were enrolled in the study. The mean latency from hospital admission to surgical intervention was 2.5 ± 3.2 days. All patients treated with surgical evacuation demonstrated postoperative clinical improvement in terms of GCS and/or MGS scores. The univariate and multivariate analyses demonstrated significantly better neurological outcomes and shorter length of hospitalization in patients treated within 3 days from hospital admission. No statistically significant associations were demonstrated between surgical timing and postoperative complication.

**Conclusions:**

This is the first study to identify a specific surgical timing cut-off in the treatment of mildly symptomatic CSDH associated with improved clinical outcomes and recovery, offering a potential reference point for clinical decision-making. Patients who underwent surgery within three days from hospital admission exhibited significantly better postoperative neurological outcomes and shorter hospital stays. Surgical timing did not influence postoperative complications, including hematoma recurrence or the need for early reintervention.

## Introduction

Chronic Subdural Hematoma (CSDH) is one of the most commonly encountered conditions in the neurosurgical practice with an overall incidence of 1.72 to 20.6 per 100,000 people per year peaking in the elderly population [1]. To date, there is still uncertainty regarding the exact pathophysiological mechanisms underlying CSDH, and multiple theories have been proposed [2–5]. The clinical presentation at onset is often heterogeneous and covers a wide spectrum of possible neurological symptoms, largely depending on intrinsic characteristics of hematoma such as volume, location, and mass effect. Glasgow Coma Scale (GCS) and Markwalder’s Neurological Grading Score (MGS) are standardized prognostic tools routinely used to assess consciousness impairment and the severity of neurological symptoms in patients with CSDH, respectively [6].

Over the past decades, several risk factors have been found to play a role in the pathogenesis of CSDH. History of previous head trauma has been identified in 50–77% of patients with newly diagnosed CSDH [5]. Other reported risk factors include anticoagulant or antiplatelet therapy, male sex, exposure to high altitudes, craniocerebral disproportion, and cerebrospinal fluid leakage [7–15]. The management of CSDH depends mainly on the severity of neurological symptoms and radiological characteristics of the hematoma, with surgical intervention indicated mostly for symptomatic cases and/or hematoma with thickness > 10 mm or midline shift > 7 mm [16]. Medical treatment with dexamethasone might be an option in selected cases when upfront surgery is not indicated, although it is associated with a higher complications rate and greater likelihood of delayed surgery [17].

To date, the role of timing in CSDH surgery in mildly symptomatic patients remains uncertain and data regarding its potential relationships with postoperative clinical outcomes are limited [18]. On this basis, the primary aim of this study was to analyze the prognostic role of surgical timing in patients with CSDH, proposing a timing cut-off potentially associated with favorable postoperative neurological outcomes after surgical treatment.

## Materials and methods

In this multicenter retrospective cohort study, patients diagnosed with mild symptomatic CSDH treated with surgical evacuation from January 2020 to December 2023 were enrolled. Only patients considered eligible for surgery by a senior surgeon based on clinical and radiological characteristics, for whom preoperative and postoperative GCS and MGS scores were available were included. Patients presenting with preoperative GCS score ≥ 13 and MGS score ≤ 2 scores were defined as “mild symptomatic”. Neurologically asymptomatic patients (preoperative GCS score 15 and MGS score 0), and patients with severe clinical presentation (preoperative GCS score < 13 or MGS > 2) were excluded from the study.

This study was conducted in accordance with the Guidelines for Good Clinical Practice and the Declaration of Helsinki (2002) of the World Medical Association. Written informed consent was regularly obtained from all patients for every diagnostic and surgical procedure.

### Study design

Patient electronic medical records were reviewed for demographic data, clinical and radiological evaluations. Comorbidities in the past medical history considered for the analysis included: history of systemic hypertension, atrial fibrillation, coronary artery disease, oncological history, chronic use of antiplatelet, anticoagulant, or antiepileptic drugs. All patients included in the study were treated with surgical evacuation by a single burr hole under sedation and regional anesthesia. External subdural drainage was placed in all patients and removed 2 days after the surgery. Perioperative antibiotic prophylaxis was regularly prescribed until drainage removal. Non-enhanced head CT scan was routinely performed 48 h after the surgery and one month after hospital discharge to evaluate the extent of hematoma evacuation and to rule out potential early or late postoperative complications. Length of hospitalization (LoH) was determined for all patients.

Neurological status was evaluated preoperatively at admission and postoperatively during the hospitalization using the Glasgow Coma Scale (GCS) and Markwalder Grading System (MGS) scores [6]. Favorable postoperative neurological outcome was defined as an improvement of at least 1 point in postoperative MGS score compared to preoperative evaluation. Surgical timing in terms of number of days from hospital admission to surgical intervention was determined in all patients. A receiver operating characteristic (ROC) curve analysis was used to identify the optimal surgical timing cut-off day related to postoperative outcome in terms of deltaMGS score (ΔMGS, defined as the difference between mean preoperative MGS and mean postoperative MGS scores). Subsequently, univariate and multivariate analysis were performed to verify potential significant associations between surgical timing and postoperative neurological outcome, length of hospitalization, and postoperative complication.

The primary objective of this study was to identify a cut-off for surgical timing and evaluate its correlation with potential favorable postoperative clinical outcomes. The secondary objective was to establish whether the timing of surgery influenced postoperative complication and length of hospitalization.

### Statistical analysis

Descriptive statistics were reported as mean and standard deviation for continuous variables or frequency and percentage for qualitative variables. Statistical significance was defined as a p-value ≤ 0.05. The Mann-Whitney U test was used to evaluate the difference in the scores of two quantitative variables. To assess the impact of one or more independent variables on a quantitative dependent variable, the univariate or multivariate linear regression model was used, depending on the number of independent variables included in the model. To assess the impact of one or more independent variables on a qualitative dependent variable, the univariate or multivariate logistic regression model was used, depending on the number of independent variables included in the model. A ROC curve analysis with Youden index determination was performed to identify the optimal surgical timing cut-off related to postoperative outcome. All statistical analyses were performed with SPSS Statistics software (IBM SPSS Statistics for Windows, version 28.0; IBM Corp., Armonk, New York, USA).

## Results

A total of 160 patients were enrolled in the study based on the inclusion and exclusion criteria. Overall, 117 (73.2%) patients were male and 43 (26.8%) were female. The mean age at the time of surgery was 77.5 ± 9.8 years (range: 49–98 years). A history of previous head trauma was globally reported in 135 (84.3%) patients. Comorbidities included systemic hypertension (102 patients, 63.7%), coronary heart disease (35 patients, 21.8%), history of oncological disease (34 patients, 21.2%), and atrial fibrillation (28 patients, 17.5%). At the time of the diagnosis, 27 (16.8%) patients were on long-term anticoagulation therapy, 48 (30%) were on long-term antiplatelet therapy, and 13 (8.1%) were on long-term antiepileptic therapy. Unilateral hematoma was observed in 124 patients (77.5%), while bilateral hematoma was present in 36 patients (22.5%).

The mean time from hospital admission to surgical intervention was 2.5 ± 3.2 days, with a median of 2 days. All patients who underwent surgical intervention demonstrated postoperative clinical improvement in terms of either GCS and/or MGS scores. The mean preoperative and postoperative GCS scores were 14.3 ± 0.6 and 14.7 ± 0.9, respectively. Mean preoperative and postoperative MGS scores were 1.4 ± 0.5 and 0.4 ± 0.6, respectively. Overall mean length of hospitalization was 7.2 ± 4.7 days. Complete demographic characteristics, as well as preoperative, postoperative, and clinical outcome data of the cohort are summarized in Table 1.

**Table 1:** Demographic characteristics, preoperative, postoperative and clinical outcome data of the cohort. GCS: Glasgow coma sale; MGS: Markwalder grading system

	*n* = 160
Sex Male Female	117 (73.2%) 43 (26.8%)
Age [mean ± SD (range min-max)]	77.5 ± 9.8 (49–98)
Comorbidities Systemic hypertension Oncological disease Coronary artery disease Atrial fibrillation	102 (63.7%) 34 (21.2%) 35 (21.8%) 28 (17.5%)
Long-term medications Antiplatelet therapy Anticoagulant therapy Antiepileptic therapy	48 (30.0%) 27 (16.8%) 13 (8.1%)
Hematoma distribution Unilateral Bilateral	124 (77.5%) 36 (22.5%)
Time from hospital admission to surgical evacuation [(days, mean ± SD (median)] Patients operated ≤ 3 days from admission Patients operated > 3 days from admission	2.5 ± 3.2 (2) 126 (78.7%) 34 (21.3%)
Clinical outcome (mean ± SD) Preoperative GCS score Postoperative GCS score Preoperative MGS score Postoperative MGS score	14.3 ± 0.6 14.7 ± 0.9 1.4 ± 0.5 0.4 ± 0.6
Overall LoH [days, mean ± SD (median)] LoH of patients operated ≤ 3 days from admission LoH of patients operated > 3 days from admission	7.2 ± 4.7 (6) 6.0 ± 3.1 (5.5) 12.8 ± 5.8 (11)
Postoperative complication Hematoma recurrence requiring surgical reintervention during hospitalization Hematoma recurrence on follow-up CT scan	12 (7.5%) 15 (9.3%)

### Impact of surgical timing on postoperative outcome

**Fig. 1:**
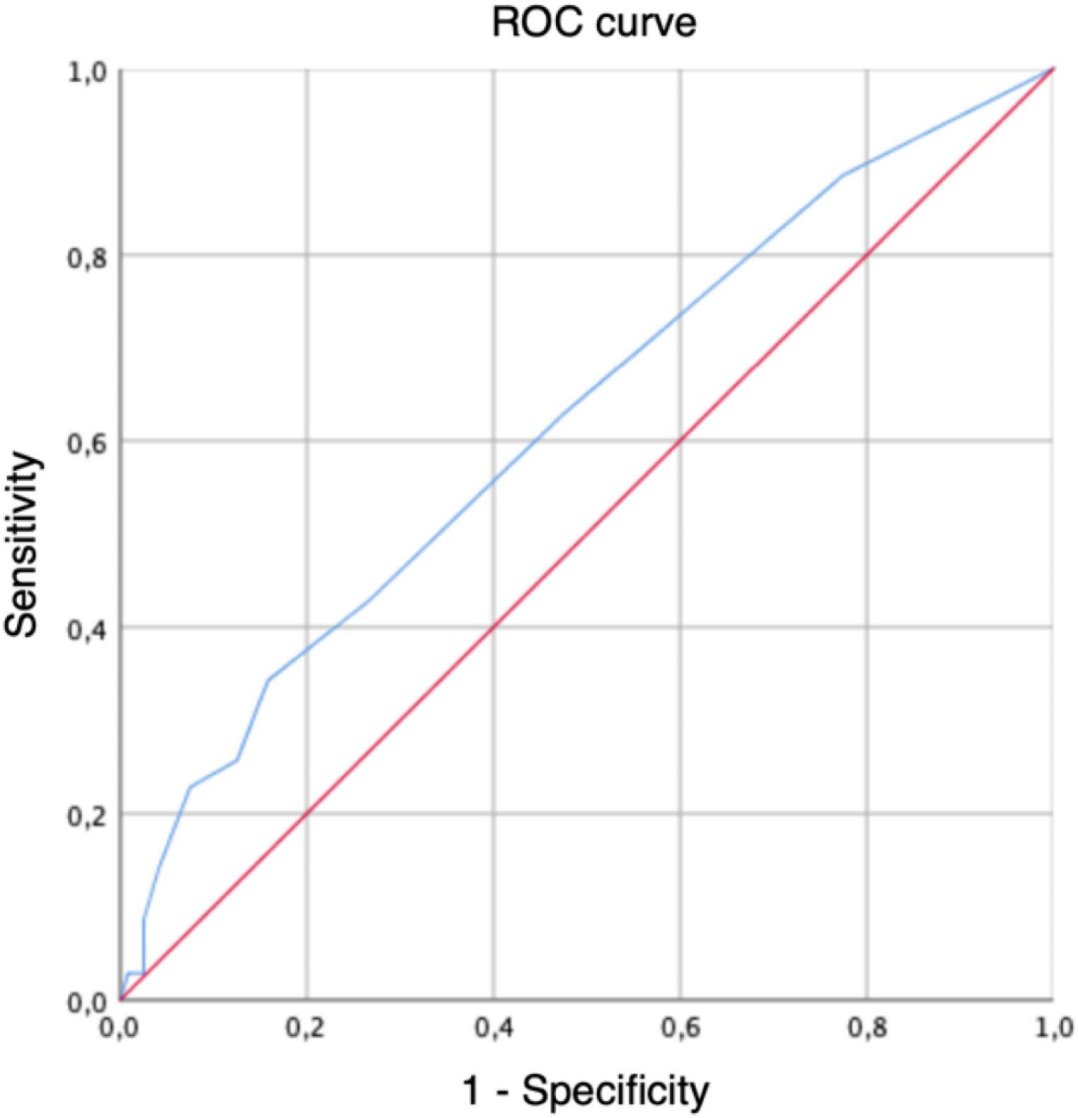
ROC curve analysis considering surgical timing and deltaMGS score

A ROC curve analysis was performed to identify a specific cut-off day for surgical timing associated with a potentially favorable postoperative clinical outcome in terms of ΔMGS scores. The analysis of Youden indices identified the optimal surgical timing cut-off day between 2 and 3 days from hospital admission to surgical intervention. Table 2; Fig. 1 provide a more detailed illustration the ROC curve analysis result. Subsequently, a univariate binary logistic regression model was used to evaluate the relationship between surgical timing and postoperative clinical outcome in terms of ΔMGS scores. In the group of patients who underwent surgery more than 3 days from hospital admission, the univariate analysis demonstrated a statistically significant 63.9% reduction in the probability of achieving postoperative favorable ΔMGS scores (*p* = 0.019).

In addition, a multivariate binary logistic regression model was performed to evaluate the relationship between surgical timing, postoperative clinical outcome in terms of ΔMGS scores, and preoperative characteristics including age, sex, preoperative GCS score, comorbidities in the past medical history, and anticoagulant, antiplatelet or antiepileptic therapies. The results of the multivariate analysis confirmed a statistically significant association between surgical timing and ΔMGS scores. More specifically, the multivariate analysis demonstrated a statistically significant 67.9% reduction in the probability of achieving postoperative favorable ΔMGS scores in the group of patients who underwent surgery more than 3 days from hospital admission (*p* = 0.022). No statistically significant results were obtained for the other variables included in the analysis. Complete data regarding univariate and multivariate logistic regression models are presented in Tables 3 and 4, respectively.

**Table 2:** ROC curve analysis description considering surgical timing and DeltaMGS score. According to Youden index, the optimal surgical timing cut-off is 3 days. AUC: area under the curve

Value	Sensitivity	1 - Specificity
− 1	1.000	1.000
0.5	0.886	0.775
1.5	0.629	0.475
**2.5**	**0.429**	**0.267**
3.5	0.343	0.158
4.5	0.257	0.125
5.5	0.229	0.075
6.5	0.143	0.042
7.5	0.086	0.025
8.5	0.029	0.025
13	0.029	0.017
18	0.029	0.008
21	0.000	0.000
AUC	0.622	

**Table 3:** Univariate binary logistic regression model evaluating the relationship between DeltaMGS score and surgical timing

*R*^2^ Nagelkerke			0.050	
	Odd Ratio	pValue	C.I. 95% Inf	C.I. 95% Sup
Surgical timing > 3 days	0.361	**0.019**	0.154	0.845

**Table 4:** Multivariate binary logistic regression model evaluating the relationship between DeltaMGS score and surgical timing

*R*^2^ Nagelkerke			0.104	
	Odd Ratio	pValue	C.I. 95% Inf	C.I. 95% Sup
Surgical timing > 3 days	0.321	**0.022**	0.121	0.849
Systemic hypertension	0.601	0.278	0.239	1.508
Atrial fibrillation	0.686	0.561	0.191	2.451
Coronary artery disease	0.869	0.818	0.262	2.877
Oncological history	0.789	0.625	0.304	2.042
Antiepileptic therapy	0.596	0.480	0.141	2.506
Antiplatelet therapy	2.028	0.237	0.628	6.539
Anticoagulant therapy	1.247	0.746	0.328	4.736
Preoperative GCS score	0.813	0.562	0.404	1.635
Age	0.989	0.640	0.944	1.036
Sex	0.583	0.213	0.249	1.363

### Impact of surgical timing on length of hospitalization and postoperative complication

A univariate binary linear regression model was used to evaluate the relationship between surgical timing and length of hospitalization. In the group of patients who underwent surgery more than 3 days from hospital admission, the univariate analysis demonstrated a statistically significant increase of 6.8 days in hospitalization (*p* < 0.001). Subsequently, a multivariate binary linear regression model was performed to evaluate the relationship between surgical timing, length of hospitalization, and preoperative characteristics including age, sex, preoperative GCS score, comorbidities in the past medical history, and anticoagulant, antiplatelet or antiepileptic therapies. The results of the multivariate analysis confirmed a statistically significant association between surgical timing and length of hospitalization. More specifically, the multivariate analysis demonstrated a statistically significant increase of 6.7 days in hospitalization in the group of patients who underwent surgery more than 3 days from hospital admission (*p* < 0.001). In addition, the multivariate analysis showed a statistically significant reduction of 1.4 days in hospitalization every 1-point increase in preoperative GCS score (*p* = 0.004). No statistically significant results were obtained for the other variables included in the analysis.

Lastly, a univariate binary logistic regression model was used to evaluate the relationship between surgical timing and postoperative complications in terms of hematoma recurrence requiring surgical reintervention during hospitalization, and hematoma recurrence on follow-up CT scan. In the group of patients who underwent surgery more than 3 days from hospital admission, no statistically significant differences were demonstrated in terms of risk of postoperative complications (*p* > 0.005). Complete data regarding univariate and multivariate regression models are presented in Tables 5 and 6, and Table 7.

**Table 5:** Univariate linear regression model evaluating the relationship between length of hospitalization and surgical timing. LoH: length of hospitalization

*R* ^2^			0.343	
	LoH increment	pValue	C.I. 95% Inf	C.I. 95% Sup
Surgical timing > 3 days	6.85	**< 0.001**	5.34	8.96

**Table 6:** Multivariate linear regression model evaluating the relationship between length of hospitalization and surgical timing

*R* ^2^			0.466	
	LoH increment	pValue	C.I. 95% Inf	C.I. 95% Sup
Surgical timing > 3 days	6.777	**< 0.001**	0.121	0.849
Systemic hypertension	0.536	0.421	0.239	1.508
Atrial fibrillation	− 1.315	0.194	0.191	2.451
Coronary artery disease	− 0.420	0.628	0.262	2.877
Oncological history	− 0.540	0.463	0.304	2.042
Antiepileptic therapy	1.981	0.081	0.141	2.506
Antiplatelet therapy	− 0.284	0.733	0.628	6.539
Anticoagulant therapy	1.074	0.301	0.328	4.736
Preoperative GCS score	− 1.478	**0.004**	0.404	1.635
Age	− 0.018	0.594	0.944	1.036
Sex	0.524	0.430	0.249	1.363

**Table 7:** Univariate logistic regression model evaluating the relationship between surgical timing and postoperative complications in terms of hematoma recurrence at follow-up CT scan and need for reintervention during hospitalization

*R*^2^ Nagelkerke– “Hematoma recurrence”			0.001	
R^2^ Nagelkerke– “Reintervention during hospitalization”			0.009	
	Odd Ratio “Hematoma recurrence “	pValue	C.I. 95% Inf	C.I. 95% Sup
Surgical timing > 3 days	1.03	0.869	0.738	1.43
	Odd Ratio “Reintervention during hospitalization”	pValue	C.I. 95% Inf	C.I. 95% Sup
Surgical timing > 3 days	1.06	0.388	0.920	1.239

## Discussion

The optimal management of CSDH is a relevant topic for neurosurgical practice. The increasing longevity of the population and the more frequent use of anticoagulant and antiplatelet therapies are expected to play a significant role in the incidence of CSDH [5, 19, 20]. Consequently, it is essential to optimize the neurosurgical holistic management of CSDH, with particular attention to surgical treatment. The choice of adequate timing for surgical evacuation is still subject of debate and often surgeon-dependent, except in the most obvious cases requiring urgent intervention. The identification of timing cut-offs for surgical intervention, along with a comprehensive preoperative assessment of patients, could contribute to achieve better postoperative outcomes and shorter hospital stays.

Although surgical time frames have been widely evaluated in acute subdural hematomas, data in the available literature on the impact of surgical timing on postoperative outcome in CSDH are limited. In a multicenter observational cohort study, Venturini et al. demonstrated a positive linear relationship between time to surgery and length of stay, even though no correlation between time to surgery and postoperative clinical outcome was observed. Nonetheless, no clear timing cut-off was established, suggesting proceeding with surgical intervention as soon as practically feasible ensuring comprehensive preoperative assessment and patient optimization [18]. In a retrospective cohort study by Zolfaghari et al., increased time from initial CT scan to surgical evacuation for CSDH did not negatively impact postoperative outcome. However, it should be noted that mean time from CT scan to surgery was 76 h, ultimately within 7 days [21]. Regarding preoperative prognostic factors other than latency of surgical evacuation, data from the literature are heterogeneous. History of renal disease and dialysis, brain lesions, anemia or thrombocytopenia, use of anticoagulant medication, need of preoperative intubation and inability to extubate the patient after evacuation are the main described prognostic factors related to poorer outcomes after surgery [22, 23]. Furthermore, data from recent metanalysis described poorer postoperative outcomes and higher rates of CSDH recurrence in patients with hyperdense and non-homogenous hematoma appearance on CT scan, and in cases treated without placement of closed system drains after evacuation [24, 25]. Finally, several attempts have been made to develop predictive models for postoperative outcomes after CSDH evacuation, yet with inadequate results [26].

The findings of this study provide new insights into the prognostic role of surgical timing in CSDH treatment, highlighting its impact on postoperative outcomes and length of hospitalization. In routine neurosurgical practice, it is common for the surgical intervention of mildly symptomatic CSDH patients to be delayed due to scheduling constraints and the need to prioritize more urgent neurosurgical cases. This practical challenge often results in prolonged hospital stays before surgery, which, as demonstrated in this study, may negatively impact overall patient outcomes. Our results demonstrate that patients who underwent surgery within three days from hospital admission had significantly better postoperative clinical outcomes, as evidenced by improvements in MGS scores. Additionally, early surgical intervention was associated with a statistically significant reduction in the length of hospitalization. The correlation between better preoperative GCS scores and shorter hospital stays emphasizes the prognostic significance of baseline neurological status, suggesting that patients with preserved consciousness at admission are more likely to experience faster postoperative recovery, potentially benefiting from an expedited discharge process. Importantly, surgical timing did not influence postoperative complications, including hematoma recurrence or the need for reintervention. These findings underscore the importance of timely surgical management in optimizing patient recovery and suggest that early intervention may contribute to improved healthcare efficiency and reduced hospital costs.

Despite its strengths, this study has some limitations. As a retrospective study, it is subject to potential selection and recall bias. The absence of a control group and a randomization protocol limits the generalizability of the results. Moreover, all patients in this study underwent surgical evacuation with a single burr-hole craniostomy, making the findings potentially inapplicable to patients treated with other surgical techniques. Further prospective studies with randomized designs are needed to validate these findings and establish definitive clinical guidelines.

## Conclusions

This is the first study to identify a specific surgical timing cut-off in the treatment of mildly symptomatic CSDH associated with improved clinical outcomes and recovery, offering a potential reference point for clinical decision-making. Patients who underwent surgery within three days from hospital admission exhibited significantly better postoperative neurological outcomes and shorter hospital stays. Surgical timing did not influence postoperative complications, including hematoma recurrence or the need for early reintervention. These results support the prioritization of early surgical intervention in eligible patients, potentially improving both clinical outcomes and healthcare resource utilization. Future prospective trials are warranted to confirm these findings and refine recommendations for the optimal timing of surgical evacuation in CSDH patients.

## Data Availability

No datasets were generated or analysed during the current study.
